# Comprehensive Association Analysis of 21-Gene Recurrence Score and Obesity in Chinese Breast Cancer Patients

**DOI:** 10.3389/fonc.2021.619840

**Published:** 2021-03-26

**Authors:** Yiwei Tong, Weiqi Gao, Jiayi Wu, Siji Zhu, Ou Huang, Jianrong He, Li Zhu, Weiguo Chen, Yafen Li, Kunwei Shen, Xiaosong Chen

**Affiliations:** Department of General Surgery, Comprehensive Breast Health Center, Ruijin Hospital, Shanghai Jiao Tong University School of Medicine, Shanghai, China

**Keywords:** breast cancer, body mass index, obesity, prognosis, recurrence score

## Abstract

**Purpose:**

A center-specific 21-gene recurrence score (RS) assay has been validated in Luminal-like, HER2-, pN0-1 Chinese breast cancer patients with both predictive and prognostic value. The association between RS and host factors such as obesity remains unclear. The objectives of the current study are to comprehensively analyze the distribution, single gene expression, and prognostic value of RS among non-overweight, overweight and obese patients.

**Patients and methods:**

Luminal-like patients between January 2009 and December 2018 were retrospectively reviewed. Association and subgroup analysis between BMI and RS were conducted. Single-gene expression in RS panel was compared according to BMI status. Disease-free survival (DFS) and overall survival (OS) were calculated according to risk category and BMI status.

**Results:**

Among 1876 patients included, 124 (6.6%), 896 (47.8%) and 856 (45.6%) had RS < 11, RS 11-25, and RS ≥ 26, respectively. Risk category was significantly differently distributed by BMI status (*P*=0.033). Obese patients were more likely to have RS < 11 (OR 2.45, 95% CI 1.38-4.35, *P*=0.002) compared with non-overweight patients. The effect of BMI on RS significantly varied according to menstruation (*P*<0.05). Compared to non-overweight patients, obese ones presented significantly higher *ER*, *PR*, *CEGP1*, *Ki67*, *CCNB1* and *GSTM1* (all *P*<0.05) mRNA expression, and such difference was mainly observed in postmenopausal population. After a median follow-up of 39.40 months (range 1.67-119.53), RS could significantly predict DFS in whole population (*P*=0.001). RS was associated with DFS in non-overweight (*P*=0.046), but not in overweight (*P*=0.558) or obese (*P*=0.114) population.

**Conclusions:**

RS was differently distributed among different BMI status, which interacted with menopausal status. Estrogen receptor and proliferation group genes were more expressed in obese patients, especially in postmenopausal population.

## Introduction

Breast cancer is the most frequent malignancy reported in women worldwide (1). About 70% of breast cancer patients are of Luminal-like, human epidermal growth factor 2 (HER2)-negative subtype, which is characterized by the expression of hormone receptor (HR), and the absence of HER2 amplification (2). Over the past decade, in addition to traditional tumor anatomic, biologic features, genetic factors have been integrated to guide treatment decisions as well as predict disease outcomes in these patients. The Oncotype Dx is the most common multigene panel to predict chemotherapy benefit and prognosis for HR-positive, HER2-negative, node-negative patients, based on the findings of the prospective TAILORx trial (3, 4). In order to facilitate the application of genetic panel in the management of Chinese breast cancer patients, a center-specific 21-gene recurrence score (RS) panel was developed based on quantitative reverse transcription-polymerase chain reaction (RT-PCR) technique. Our RS panel has previously been validated in both node-negative (5) and node-positive (6) patients with two large cohorts of Chinese patients. Increased RS was associated with poor differentiation, PR-negative or high-proliferation characteristics in Chinese early breast cancer patients, as indicated in our former work (5), which was comparable to the findings for Oncotype Dx in NSABP B-14 study population (4). In addition, our RS panel showed similar prognostic value in node-negative and positive diseases (6). With the help of RS testing, selective low RS patients can be spared from adjuvant chemotherapy, while chemotherapy is recommended for high RS patients.

Apart from tumor-intrinsic factors, the microenvironment in which tumors arise and progress substantially varies between individuals, calling for the necessity to identify host determinants for tumor behaviors (7). Obesity is a well-established risk factor for multiple cancers including breast cancer (7, 8). The evidence for the effect of obesity on breast cancer is generally based on studies using body mass index (BMI) as an alternative for total adiposity (9). The effect of obesity and overweight on breast cancer incidence differs before and after menopause (8). Several large meta‐analyses have showed an inverse association between obesity and breast cancer risk in premenopausal population, with breast cancer risk being reduced by 8% per 5 kg/m^2^ BMI increase (8–11). On the other hand, for postmenopausal women, obesity is positively associated with both increased overall and increased HR-positive breast cancer risk (8, 10). With regards to clinical outcomes, obesity is related to higher risk of disease recurrence and mortality for both premenopausal and postmenopausal breast cancer, with every 5 kg/m^2^ increase in BMI augmenting the risk of breast cancer-specific death by 18% (8).

However, how obesity or overweight interacts with patient genetic profiles remains uncertain for breast cancer patients. In a retrospective study including 534 women with HR-positive, HER2-negative disease, Muniz et al. found that neither metabolic syndrome, nor any individual criterion including central obesity, had significant association with 21-gene RS group after stratification by menstrual status (12). It is also unclear whether RS can accurately predict disease outcomes in patients with different BMI status. One retrospective study involving 940 HR-positive breast cancer patients from the transATAC trial showed that Oncotype Dx had the highest prognostic effect in patients with BMI ≤ 25 kg/m^2^, but decreasing effect size with increasing BMI (13). Evidence is still limited with regards to the relationship between genetic risk score and host BMI status in Luminal-like patients.

Therefore, in the current study, we aim to analyze the distribution of RS category and gene expression level among non-overweight, overweight and obese Chinese patients, to identify potential impact factors for the association of RS and BMI in HR-positive, HER2-negative breast cancer patients, and to explore the prognostic value of RS in Chinese patients with different BMI status.

## Materials and Methods

### Study Population

Consecutive breast cancer patients receiving surgery in Comprehensive Breast Health Center, Ruijin Hospital, Shanghai Jiao Tong University School of Medicine, Shanghai, China, between January 2009 to December 2018 were retrospectively reviewed. The inclusion criteria were as listed below: 1) female gender; 2) invasive breast cancer; 3) HR-positive, HER2-negative disease; 4) available 21-gene RS result with cycle threshold (C_T_) values for each gene. Exclusion criteria were as follows: 1) patients receiving preoperative systemic treatment; 2) *de novo* stage IV disease. The current study was reviewed and approved by the independent Ethical Committees of Ruijin Hospital, Shanghai Jiao Tong University School of Medicine. Written informed consent was obtained from each participant. All procedures were in accordance with the ethical standards of national research committee and with the 1964 Helsinki declaration and its later amendments.

### Data Collection

Patient clinical information was retrieved from Shanghai Jiao Tong University Breast Cancer Database (SJTU-BCDB). Patients aged no less than 60 years, <60 years and amenorrheic for ≥ 36 months, or with prior bilateral oophorectomy were considered postmenopausal. Patient’s height and weight were measured on the day of hospital admission for surgical treatment and BMI was calculated by dividing weight (kg) by the square of height (m^2^). Patients were then classified into non-overweight (BMI <24.0 kg/m^2^), overweight (BMI ≥ 24.0 and <28.0 kg/m^2^) and obese (BMI ≥ 28.0 kg/m^2^) subgroups, according to the recommended cutoffs for Chinese population from the Guidelines for Prevention and Control of Overweight and Obesity in Chinese Adults (Ministry of Health of the People’s Republic of China, People’s Medical Publishing House, 2006) (14, 15).

The histo-pathologic evaluation of the tumor was accomplished in the Department of Pathology, Ruijin Hospital by at least two independent, experienced pathologists (C Wang, X Fei, X Jin and J Xie). The American Society of Clinical Oncology/College of American Pathologists (ASCO/CAP) guidelines were adopted for the immunohistochemistry (IHC) assessment of estrogen receptor (ER), progesterone receptor (PR), HER2 and Ki-67, as described in our previous studies (5, 6). HR positive was defined as no less than 1% invasive tumor cells with positive nuclear staining (16). The cut-off point for ER high and low expression was set at 50% (17, 18). HER2 negative was defined as IHC 0 or 1+, and IHC 2+ with fluorescence *in situ* hybridization negative (19). According to the 2013 St. Gallen Consensus, tumors were classified into two molecular subtypes, which were Luminal A-like (ER+/PR≥20%/Ki-67<14%), and Luminal B-like (ER-/PR+/any Ki-67, or ER+/PR<20%/any Ki-67, or HR+/Ki-67≥14%) (18).

Patient follow-up was accomplished by specialized breast cancer nurses in our center. Clinical outcomes were analyzed according to the STEEP system (20). Disease-free survival (DFS) was calculated from the date of surgery to the recurrence of tumor including ipsilateral, local/regional or distant recurrence, second non-breast malignancy, and death attributable to any cause. Overall survival (OS) was calculated from the date of surgery till death of any cause. Last follow-up was completed by February 2020.

### 21-Gene Recurrence Score Evaluation

The 21-gene assay testing was conducted in the Department of Clinical Laboratory, Ruijin Hospital by Lin L, Lin J and Meng J, as described in our previous work (5, 6). RNA extraction and reverse transcription were performed with RNeasy FFPE RNA kit (Qiagen, 73504, Germany) and Omniscript RT kit (Qiagen, 205111, Germany), respectively. Quantitative RT-PCR was accomplished in Applied Biosystems 7500 Real-Time PCR System (Foster City, CA) using Premix Ex TaqTM (TaKaRa Bio, RR390A). C_T_ value, defined as the number of cycles required for the fluorescent signal to cross a certain threshold, was verified in triplicate, and then normalized to reference genes β-*actin*, *GAPDH*, *GUS*, *RPLPO* and *TFRC*. The relative expression level of each target gene, in form of -ΔC_T_ value, was defined as C_T reference_ -C_T gene_. The 21-gene RS was calculated from the reference gene-normalized formula, then applied to classify patients into low risk (RS ≤ 11), intermediate risk (RS 11-25), and high risk (RS ≥ 25) groups. For those with multifocal diseases, the highest RS was recorded.

### Statistical Analysis

Chi-square test and multivariate logistic regression were applied to compare the distribution of categorical variables by BMI status in the study population. T-test was adopted to compare the distribution of RS by BMI intervals. Subgroup analysis of interacting factors with BMI and 21-gene RS was accomplished using stratified Mantel-Haenszel test to estimate odds ratio (OR) with 95% confidence interval (CI). The comparison of gene expression in 21-gene RS panel by BMI status was demonstrated in terms of violin plots. Univariate survival analyses were conducted using Kaplan–Meier curves. Data analysis and image production were performed using IBM SPSS statistics software version 23 (SPSS, Inc., Chicago, IL) and GraphPad Prism version 8.0 (GraphPad Software, CA, USA). Two-sided *P* value <0.05 was considered statistically significant.

## Results

### Baseline Characteristics Stratified by Body Mass Index Status

Overall, 1876 Luminal-like breast cancer patients were enrolled in the current study (**Supplementary Figure S1**). The baseline clinical pathological characteristics of the participants were presented in **Table 1**. The average age was 57 ± 12.50 (range 24-92) years. All but four patients had ER-positive disease, among whom 98 had ER ≤ 50%. PR staining was positive in 88.2% of the population. Luminal A-like and Luminal B-like subtypes were found in 32.0% and 68.0% cases.

**Table 1:** Baseline characteristics of study participants (N = 1,876).

Characteristics	Total	Non-overweight	Overweight	Obese	*P* value
	N = 1,876	N = 1,139 (%)	N = 551 (%)	N = 186 (%)	
Age, years					<**0.001**
<50	573	436 (38.3)	110 (20.0)	27 (14.5)	
50-65	812	451 (39.6)	271 (49/2)	90 (48.4)	
>65	491	252 (22.1)	170 (30.9)	69 (37.1)	
Menopausal status					<**0.001**
Premenopausal	654	482 (42.3)	138 (25.0)	34 (18.3)	
Postmenopausal	1222	657 (57.7)	413 (75.0)	152 (81.7)	
Breast surgery					0.470
BCS	827	515 (45.2)	233 (42.3)	79 (42.5)	
Mastectomy	1049	624 (54.8)	318 (57.7)	107 (57.5)	
ALN surgery					**0.027**
SLNB	879	562 (49.3)	237 (43.0)	80 (43.0)	
ALND	997	577 (50.7)	314 (57.0)	106 (57.0)	
Histology					0.434
IDC	1616	977 (85.8)	473 (85.8)	166 (89.2)	
Non-IDC	260	162 (14.2)	78 (14.2)	20 (10.8)	
Tumor grade					**0.014**
I	175	123 (10.8)	31 (5.6)	21 (11.3)	
II	1122	667 (58.6)	339 (61.5)	116 (62.4)	
III	369	215 (18.9)	120 (21.8)	34 (18.3)	
NA	210	134 (11.8)	61 (11.1)	15 (8.1)	
Tumor size, cm					<**0.001**
≤2.0	1303	831 (73.0)	345 (62.6)	130 (69.9)	
>2.0	570	308 (27.0)	206 (37.4)	56 (30.1)	
ALN					**0.030**
Negative	1563	968 (85.0)	440 (79.9)	155 (83.3)	
Positive	313	171 (15.0)	111 (20.1)	31 (16.7)	
ER, %					0.078
≥50	1778	1069 (93.9)	531 (96.4)	178 (95.7)	
<50	98	70 (6.1)	20 (3.6)	8 (4.3)	
PR status					**0.026**
Negative	222	152 (13.3)	56 (10.2)	14 (7.5)	
Positive	1654	987 (86.7)	495 (89.8)	172 (92.5)	
Ki-67, %					0.346
<14	886	549 (48.2)	246 (44.6)	91 (48.9)	
≥14	990	590 (51.8)	305 (55.4)	95 (51.1)	
Molecular subtype					0.204
Luminal A-like	601	362 (31.8)	169 (30.7)	70 (37.6)	
Luminal B-like	1275	777 (68.2)	382 (69.3)	116 (62.4)	
21-gene RS^a^					**0.002**
Low risk	124	67 (5.9)	34 (6.2)	23 (12.4)	
Intermediate risk	896	529 (46.4)	270 (49.0)	97 (52.2)	
High risk	856	543 (47.7)	247 (44.8)	66 (35.5)	

a The cut-off for RS category was <11, 11–25, >25.

BCS, breast conserving surgery; ALN, axillary lymph node; SLNB, sentinel lymph node biopsy; ALND, axillary lymph node dissection; IDC, invasive ductal carcinoma; NA, not available; ER, estrogen receptor; PR, progesterone receptor; RS, recurrence score. Bold values mean statistically significant.

Among patients included, 1139 (60.7%) patients were non-overweight, including 84 underweight (BMI ≤ 18.5 kg/m^2^), while 551 (29.4%) were overweight, and 186 (9.9%) obese. Univariate analysis (**Table 1**) and multivariate analysis (**Table 2**) demonstrated that the overall distribution of age (*P*=0.005), grade (*P*=0.030), tumor size (*P*=0.009), and PR status (*P=*0.041) were significantly distinguishable among three BMI subgroups. Compared to non-overweight patients, overweight ones were less likely to be young (≤50 *vs >*65: OR 0.43, 95% CI 0.26-0.74, *P*<0.001), to have low tumor grade (I *vs* III: OR 0.48, 95% CI 0.30-0.78, *P*=0.003), smaller tumor size(≤2.0 *vs >*2.0: OR 0.68, 95% CI 0.54-0.87, *P*=0.002), and negative PR status (OR 0.68, 95% CI 0.47-0.98, *P*=0.037). Meantime, obese patients were less likely to be <50 years (*vs >*65: OR 0.33, 95% CI 0.14-0.76, *P*=0.009), while tumor grade, size and PR status were similarly distributed compared to non-overweight ones.

**Table 2:** Multivariate analysis of factors associated with BMI status.^a^

Characteristics	Overweight (N = 551)		Obese (N = 186)		*P* value
	OR	95% CI	OR	95% CI	
Age, years					**0.005**
<50 *vs >*65	0.43	0.26-0.74	0.33	0.14-0.76	
50-65 *vs >*65	0.91	0.69-1.19	0.80	0.55-1.16	
Menstruation status					0.413
pre- *vs* post-	0.91	0.58-1.42	0.63	0.31-1.28	
Grade					**0.030**
I *vs* III	0.48	0.30-0.78	0.90	0.48-1.67	
II *vs* III	0.91	0.69-1.20	0.96	0.62-1.47	
Tumor size, cm					**0.009**
≤2.0 *vs >*2.0	0.68	0.54-0.87	0.87	0.60-1.26	
ALN status					0.529
Negative *vs* Positive	0.88	0.66-1.18	1.12	0.72-1.76	
PR status					**0.041**
Negative *vs* Positive	0.68	0.47-0.98	0.59	0.33-1.084	
RS category^b^					**0.033**
Low *vs* High	1.05	0.65-1.70	2.45	1.38-4.35	
Intermediate *vs* High	1.13	0.89-1.43	1.40	0.97-2.03	

a The reference category for subtype characteristics is BMI < 24 kg/m^2^ (N = 1,139).

b The cutoff for RS category was <11, 11–25, >25.

BMI, body mass index; OR, odds ratio; CI, confidence interval; ALN, axillary lymph node; PR, progesterone receptor; RS, recurrence score. Bold values mean statistically significant.

### Association Between 21-Gene Recurrence Score and Body Mass Index Status

**Figure 1:**
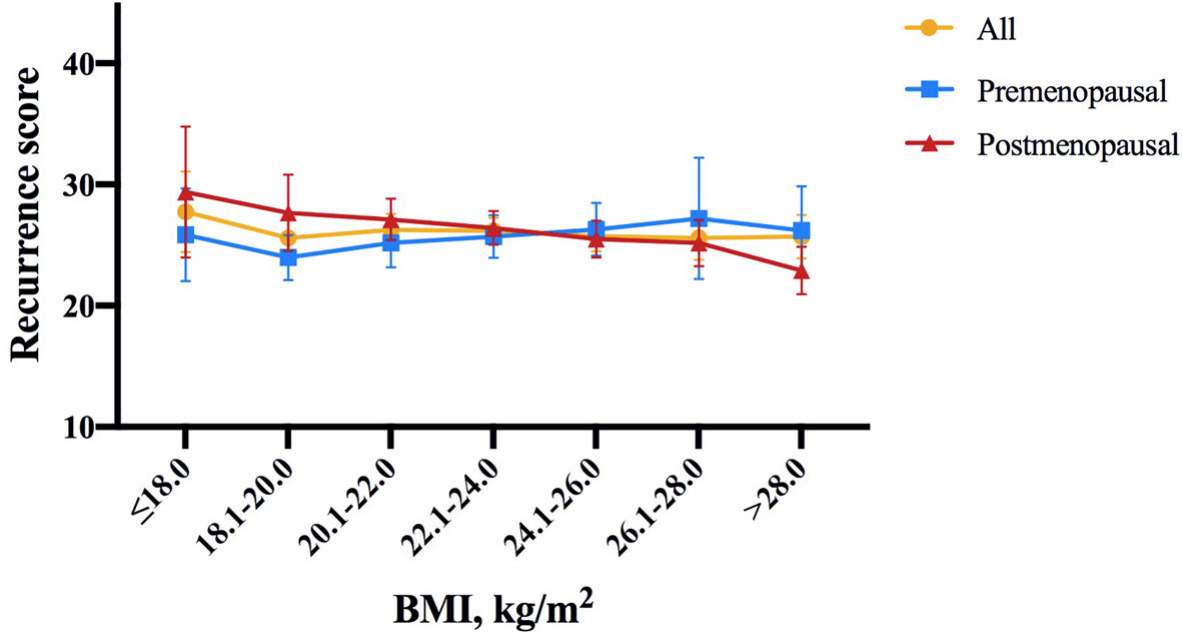
Distribution of 21-gene RS by BMI intervals of 2.0 according to menopausal status in the study population. In whole population, RS distribution was significantly different in patients with different BMI status (*P* = 0.006). Average RS score tended to decrease with increasing BMI in postmenopausal patients (*P* = 0.020), but not in premenopausal patients (*P* = 0.843). The symbols refer to average score, error bars refer to standard deviation (yellow: all patients, blue: premenopausal population, red: postmenopausal population). RS, recurrence score; BMI, body mass index.

Among the included population, 124 (6.6%), 896 (47.8%) and 856 (45.6%) were classified into low, intermediate and high risk groups, with an average 21-gene RS of 25.77 (95% CI 25.20-26.33; **Table 1**). As shown in **Figure 1**, RS distribution was significantly different in patients with various BMI status (*P*=0.006), with an average RS of 28.48 ± 11.91 in underweight, 26.05 ± 11.74 in non-overweight, 25.15 ± 11.59 in overweight, and 23.17 ± 12.26 in obese population. After adjusting for clinico-pathologic confounders, multivariate analysis demonstrated that RS category was independently significantly associated with BMI status (*P*=0.033, **Table 2**). Obese patients were more likely to have an RS ≤ 11 (OR 2.45, 95% CI 1.38-4.35, *P*=0.002) compared with non-overweight patients.

**Figure 2:**
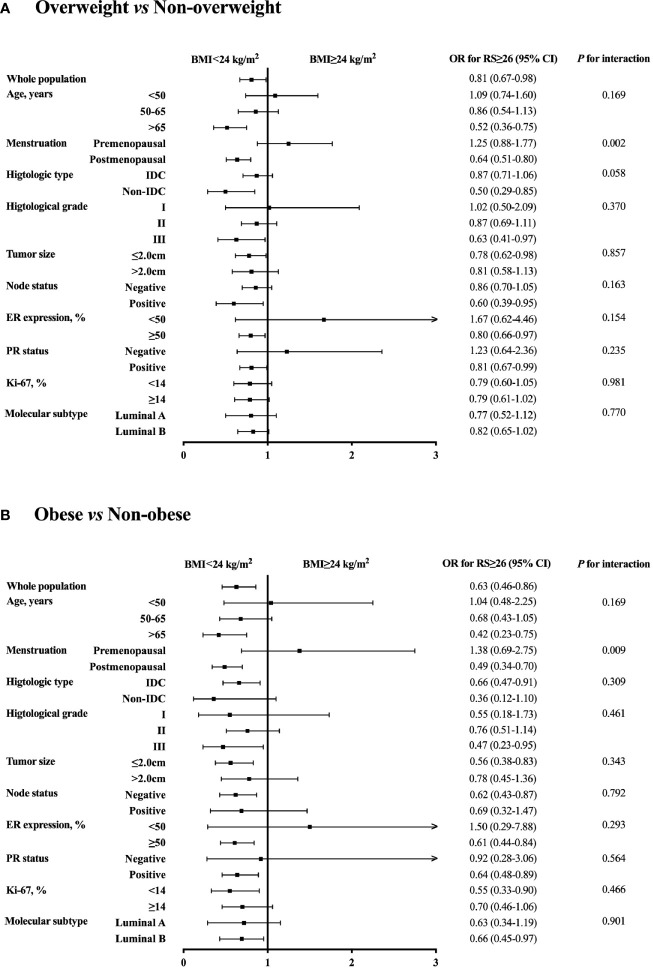
Subgroup analysis of interacted factors with BMI and 21-gene RS. The odds with 95% CI for RS ≥ 26 were compared between **(A)** overweight *vs* non-overweight, and **(B)** obese *vs* non-obese patients by each subgroup. BMI, body mass index; OR, odds ratio; RS, recurrence score; CI, confidence interval; IDC, invasive ductal carcinoma; ER, estrogen receptor; PR, progesterone receptor.

Further subgroup analysis was conducted comparing the odds of having higher RS (RS ≥ 26) between different BMI status, which identified menstruation status as the only interacting factor on the association of BMI and 21-gene RS (**Figure 2**). In detail, overweight patients were significantly less likely to have RS ≥ 26 compared to those non-overweight after menopause (OR 0.64, 95% CI 0.51-0.80, *P*<0.001), while such difference no longer held in premenopausal population (OR 1.25, 95% CI 0.88-1.77, *P*=0.216; *P* for interaction=0.002; **Figure 2A**). Alternatively, obese patients had lower odds for high risk RS than non-obese ones, but the significance was only observed in postmenopausal subgroup (OR 0.49, 95% CI 0.34-0.70, *P*<0.001), not in premenopausal women (OR 1.38, 95% CI 0.69-2.75, *P*=0.366; *P* for interaction=0.009; **Figure 2B**). In addition, average RS score decreased with increasing BMI in postmenopausal patients (*P*=0.020, **Figure 1**), but not in premenopausal patients (*P*=0.843).

### Single Gene Expression in 21-Gene Recurrence Score Panel by Body Mass Index Status

**Figure 3:**
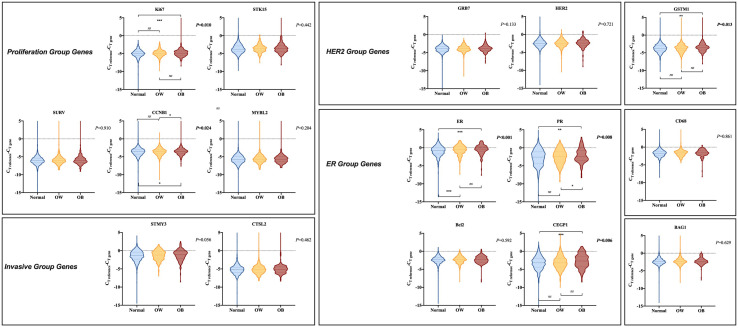
Comparison of gene expression in 21-gene RS panel by BMI status. The violin plots refer to the expression of a certain gene. The dashed lines and the dotted lines refer to the median and quartiles, respectively. BMI, body mass index; C_T_, cycle threshold; OW, overweight; OB, obese; HER2, human epidermal growth factor receptor 2; ER, estrogen receptor.

**Supplementary Table S1** summarized the gene expression and gene group score in the 21-gene RS panel of the study population. Single gene expression was further compared according to BMI status. Compared to normal weight patients, obese patients presented significantly higher ER group score (*P*=0.002), with higher *ER* (*P*<0.001; **Figure 3**), higher *PR* (*P*=0.004), higher *CEGP1* (*P*<0.001) expression, and tended to have higher proliferation group score (*P*=0.060), with higher *Ki67* (*P*=0.006), and higher *CCNB1* (*P*=0.020). In addition, *GSTM1* was significantly elevated in the obese group (*P*=0.001). On the other hand, overweight patients had generally similar gene expression compared to non-overweight ones, except for significantly higher *ER* (*P*<0.001). The HER2 group score (*P*=0.467) and invasion group score (*P*=0.210) were comparable among BMI groups.

When stratified by menstrual status, no significant difference in single gene expression among different BMI subgroups was observed in premenopausal population (**Supplementary Figure S2**). ER group (*P*=0.814), HER2 group (*P*=0.826), proliferation group (*P*=0.539), and invasion group (*P*=0.386) scores were comparable by BMI status. However, for postmenopausal population, ER group (*P*<0.001) and proliferation group (*P*=0.044) scores were significantly distinguishable among various BMI status, while HER2 group (*P*=0.252) and invasion group (*P*=0.892) scores were identical. BMI ≥ 28 kg/m^2^ was associated with considerably higher *PR* (*P*<0.001; **Supplementary Figure S3**), higher *CEGP1* (*P*<0.001), and higher *GSTM1* (*P*=0.005) expression compared to normal weight group. Overweight patients expressed higher *GRB7* (*P*=0.033) and higher *PR* (*P*<0.001) than those with BMI ≤ 24 kg/m^2^.

### Clinical Outcomes by Body Mass Index Status and Recurrence Score Category

After a median follow-up of 39.40 months (range 1.67-119.53), 109 (5.81%) DFS events were observed, including 22 local regional recurrences, 13 contralateral breast cancer, 27 distant metastases, 25 second non-breast malignancy, and 22 deaths.

In all, non-overweight, overweight, and obese patients had similar DFS in whole population (5-year DFS 91.61% *vs* 92.60% *vs* 89.41%, *P*=0.227; **Supplementary Figure S4**), low RS group (5-year DFS 95.36% *vs* 100.00% *vs* 83.33%, *P*=0.225), intermediate RS group (5-year DFS 93.70% *vs* 94.01% *vs* 94.88%, *P*=0.996), and high RS group (5-year DFS 88.97% *vs* 89.91% *vs* 83.45%, *P*=0.090), respectively. OS was also comparable among different BMI subgroups in the whole population (*P*=0.178), low RS (*P*=0.167), intermediate RS (*P*=0.809), and high RS (*P*=0.331) groups. In addition, when applying different BMI cutoff values, we also found comparable disease outcomes between patients with BMI ≥ 24 kg/m^2^ and <24 kg/m^2^ (DFS: *P*=0.933; OS: *P*=0.104; **Supplementary Figures S5A, S5B**), between patients with BMI ≥ 28 kg/m^2^ and <28 kg/m^2^ (DFS: *P*=0107; OS: *P*=0.138; **Supplementary Figures S5C, S5D**) and between patients with BMI ≥ 30 kg/m^2^ and <30 kg/m^2^ (DFS: *P*=0.198; OS: *P*=0.231; **Supplementary Figures S5E, S5F**).

**Figure 4:**
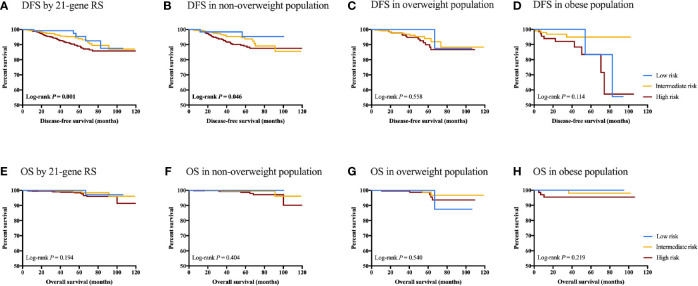
Clinical outcomes of breast cancer patients with different RS category stratified by BMI status. DFS in **(A)** DFS by 21-gene RS; **(B)** DFS in non-overweight population; **(C)** DFS in overweight population; **(D)** DFS in obese population; **(E)** OS by 21-genes RS; **(F)** OS in non-overweight population; **(G)** OS in overweight population; and **(H)** OS in obese population.

RS category significantly predicts DFS in the whole population (5-year DFS 95.41% for low RS *vs* 93.90% for intermediate RS *vs* 88.94% for high RS, *P*=0.001; **Figure 4**). Other impact factors on DFS identified in the univariate analysis included tumor grade, size, ER, Ki-67, molecular subtype, and adjuvant endocrine therapy usage (all *P*<0.05; **Supplementary Table S2**). In patients with normal weight, RS category (5-year DFS 95.36% *vs* 93.70% *vs* 88.97%, *P*=0.046), together with histology, tumor grade, size, and adjuvant endocrine therapy (all *P*<0.05) was associated with DFS. However, RS category was not associated with DFS in overweight (*P*=0.558) or obese (*P*=0.114) population. Furthermore, no statistically significant difference was found with regards to OS in the whole population (*P*=0.194), non-overweight (*P*=0.404), overweight (*P*=0.530) or obese (*P*=0.219) patients.

## Discussion

In this study, which involved 1876 HR-positive, HER2-negative breast cancer patients with 21-gene RS records, we found that RS category was significantly differently distributed among patients with different BMI status. Menstrual status was an interacting factor for the association of BMI and 21-gene RS. With regards to the gene expression of 21-gene RS panel, obese patients presented significantly higher *ER*, *PR*, *CEGP1*, *Ki67*, *CCNB1* and *GSTM1* (all *P*<0.05) than non-overweight ones in the whole population. Overweight patients had generally similar gene expression pattern except for higher *ER* (*P*<0.001) than non-overweight ones. In terms of disease outcome, BMI was not an independent factor for DFS (*P*=0.227) or OS (*P*=0.178) in HR-positive, HER2-negative patients. The prognostic value of RS was decreased in patients overweight or obese. To our knowledge, this is the largest study, as well as the first in Chinese population, to focus on the comprehensive association of 21-gene RS and BMI in Luminal-like patients, which provides evidence of association between tumor genetic profile and host metabolic factor.

Previous studies have shown that obesity leads to increased free fatty acid release, hyperinsulinemia, persistent low-grade inflammation, and abnormal secretion of adipokines, resulting in disease development or progression (8). Overwhelming consensus has been made with regards to the adverse effects of obesity on breast cancer prognosis (8, 21). For HR‐positive patients, an analysis from the NSABP B‐14 trial demonstrated that obese women had a 30% increased mortality risk compared to non-obese ones (22). In a joint analysis of 6885 women from E1199, E5188, and E3189 clinical trials, Sparano et al. showed that BMI ≥ 30 kg/m^2^ was associated with inferior DFS and OS in Luminal-like patients (23). Another meta-analysis of 21 trials indicated that obesity was associated with higher breast cancer‐specific mortality for HR-positive patients, regardless of menopausal status (24). In spite of prior studies showing impaired prognosis with obesity in breast cancer patients, our study demonstrated no significant difference in time to recurrence or mortality based on BMI status. Such discrepancy may be attributed to the different study population, different BMI cutoffs, the overall low event incidence in our cohort, and rather inadequate follow-up of 39.40 months. Moreover, when applying different BMI cutoff values, we also found comparable disease outcomes between patients with BMI ≥ 24 kg/m^2^ *vs <*24 kg/m^2^, BMI ≥ 28 kg/m^2^ *vs <*28 kg/m^2^, and BMI ≥ 30 kg/m^2^ *vs <*30 kg/m^2^. Along with our finding, one study of Cespedes Feliciano et al. found that among women with PAM50 Luminal A disease, those who had BMI ≥35 kg/m^2^, but not BMI 30-35 kg/m^2^ or overweight, had worse prognosis, while no association between BMI and prognosis was observed for Luminal B subtype (25).

The association between host obesity and genetic profile of Luminal-like breast cancer patients remains indeterminate. Several studies found a limited correlation between obesity and breast cancer genomics. For example, Muniz et al. revealed that central obesity was not associated with 21-gene RS category after stratification by menopausal status in a cohort of 534 HR-positive, HER2-negative patients (12). A lifestyle study of MINDACT trial-enrolled population involving 1555 patients showed that BMI was not an independent impact factor for the prognostic 70-gene expression signature MammaPrint, regardless of menstrual status (26). Nevertheless, our cohort of 1876 HR-positive, HER2-negative patients revealed a significant association between BMI status and RS category, which was only established in postmenopausal population. The inconsistency between previous findings and ours may be due to the BMI cutoff as well as the RS cutoff applied in the study. To note, one strength of our study was that we managed to conduct a subgroup analysis, showing that the association of BMI and RS was substantially influenced by menopausal status. After menopause, the possibility for obese or overweight patients to have a lower RS than non-overweight ones significantly rose. This is to our knowledge the first study presenting the interaction between menstruation and the correlation of BMI and 21-gene RS.

Another highlight was that we revealed, for the first time, the potential influence of BMI on single gene expression in the 21-gene RS panel. Overall, ER group genes including *ER*, *PR*, *CEGP1*, had substantially higher expression in obese patients compared to non-overweight ones, which was mainly found in the postmenopausal population. Overweight patients also expressed higher level of *ER* than those non-overweight. This finding added to the previous notion that for postmenopausal women, obesity is correlated with higher plasma levels of estradiol derived from adipose tissue (27) and increased risk of ER-positive breast cancer (28). In addition, we also found that the expression of proliferation group genes *Ki67* and *CCNB1* was significantly elevated in obese population. Meanwhile, the difference in proliferation group gene expression was less obvious between overweight *vs* non-overweight patients. As shown by Kwan et al., patients with BMI ≥ 35 kg/m^2^ had higher expression of proliferation genes compared with normal weight women (29). This might be due to the link between obesity and pro-inflammatory microenvironment, insulin resistance, the abnormal activation of insulin-like growth factor pathway, and altered adipokines, which results in more aggressive behavior of breast tumors.

Another issue to resolve is whether RS has identical prognostic value in patients with different BMI status. Sestak et al. found that 21-gene RS was most predictive in the lowest BMI tertile, and significantly less predictive in obese women, indicating an interaction of BMI and RS on the prediction of disease outcomes (13). Meanwhile in the same cohort, the prognostic value of Prosigna Risk of Recurrence Score was the greatest for women with a BMI 25 to 30 kg/m^2^ (13), suggesting that the effect of BMI on genetic assay varied across panels. In consistent with previous evidence, here we demonstrated that RS category significantly predict DFS in the whole population (*P*=0.001), and for non-overweight patients (*P*=0.046). RS category was not associated with clinical outcomes in overweight (*P*=0.558) or obese (*P*=0.114) population. The prognostic value of RS might be decreased in patients overweight or obese, but our results should be validated with longer follow-up and more events.

Apart from the strengths, there are still some limitations. First of all, given the retrospective design of the study, selection biases might be inevitable. Secondly, as a result of inadequate follow-up time and relatively superior disease outcomes, limited events were observed, so that our findings on clinical outcomes should be further validated. In addition, the current study was carried out in Chinese population, and the optimal BMI and RS cutoffs should be tested in the future to gain a better understanding of the association of obesity and 21-gene RS.

In conclusion, 21-gene RS category and gene expression were significantly differently distributed among patients with various BMI status, especially in postmenopausal patients. The prognostic value of RS might be influenced by host obesity, which warranted further validation.

## Data Availability Statement

Datasets are available on reasonable request from the corresponding authors.

## Ethics Statement

The studies involving human participants were reviewed and approved by the Ethical Committees of Ruijin Hospital, Shanghai Jiaotong University School of Medicine. The patients/participants provided their written informed consent to participate in this study.

## Author Contributions

YT analyzed and interpreted the patient data and was a major contributor in writing the manuscript. WG and JW contributed to the data collection of 21-gene recurrence score. SZ, OH, JH, LZ, WC, and YL were responsible for the clinical data collection. XC substantially contributed to the conception of the work and revised the manuscript. KS substantively revised the manuscript. All authors contributed to the article and approved the submitted version.

## Funding

The authors appreciated the financial support from the National Natural Science Foundation of China (Grant Number: 81772797), Shanghai Municipal Education Commission—Gaofeng Clinical Medicine Grant Support (20172007), Ruijin Hospital, Shanghai Jiao Tong University School of Medicine—“Guangci Excellent Youth Training Program” (GCQN-2017-A18). All these financial sponsors had no role in the study design, data collection, analysis, or interpretation.

## Conflict of Interest

The authors declare that the research was conducted in the absence of any commercial or financial relationships that could be construed as a potential conflict of interest.

## Supplementary Material

Supplementary Figure S1 Study population flowchart. HER2, human epidermal growth factor receptor 2; RS, recurrence score; CT, cycle threshold.

Supplementary Figure S2 Comparison of gene expression in 21-gene RS panel by BMI status in premenopausal population. The violin plots refer to the expression of a certain gene. The dashed lines and the dotted lines refer to the median and quartiles, respectively. BMI, body mass index; CT, cycle threshold; OW, overweight; OB, obese; HER2, human epidermal growth factor receptor 2; ER, estrogen receptor.

Supplementary Figure S3 Comparison of gene expression in 21-gene RS by BMI status in postmenopausal population. The violin plots refer to the expression of a certain gene. The dashed lines and the dotted lines refer to the median and quartiles, respectively. BMI, body mass index; CT, cycle threshold; OW, overweight; OB, obese; HER2, human epidermal growth factor receptor 2; ER, estrogen receptor.

Supplementary Figure S4 Clinical outcomes of breast cancer patients with different BMI statuses stratified by 21-gene RS category. DFS in **(A)** whole, **(B)** low risk, **(C)** intermediate risk, and **(D)** high risk population; OS in **(E)** whole, **(F)** low risk, **(G)** intermediate risk, and **(H)** high risk population. Navy, yellow and red lines refer to non-overweight, overweight and obese group, respectively. DFS, disease-free survival; OS, overall survival; BMI, body mass index; RS, recurrence score.

Supplementary Figure S5 Clinical outcomes of breast cancer patients with different BMI cutoffs. Disease-free survival and overall survival **(A, B)** whole, **(B)** low risk, **(C)** intermediate risk, and **(D)** high risk population; OS in **(E)** whole, **(F)** low risk, **(G)** intermediate risk, and **(H)** high risk population.

## References

[1] Bray F Ferlay J Soerjomataram I Siegel RL Torre LA Jernal A. Global cancer statistics 2018: GLOBOCAN estimates of incidence and mortality worldwide for 36 cancers in 185 countries. CA Cancer J Clin (2018) 68(6):394–424. 10.3322/caac.21492 30207593

[2] Turner NC Neven P Loibl S Andre F. Advances in the treatment of advanced oestrogen-receptor-positive breast cancer. Lancet (2017) 389(10087):2403–14. 10.1016/S0140-6736(16)32419-9 27939057

[3] Sparano JA Gray RJ Makower DF Pritchard KI Albain KS Hayes DF. Adjuvant Chemotherapy Guided by a 21-Gene Expression Assay in Breast Cancer. N Engl J Med (2018) 379(2):111–21. 10.1056/NEJMoa1804710 PMC6172658 29860917

[4] Paik S Shak S Tang G Kim C Baker J Cronin M. A Multigene Assay to Predict Recurrence of Tamoxifen-Treated, Node-Negative Breast Cancer. N Engl J Med (2004) 351:2817–26. 10.1056/NEJMoa041588 15591335

[5] Wu J Fang Y Lin L Fei X Gao W Zhu S. Distribution patterns of 21-gene recurrence score in 980 Chinese estrogen receptor-positive, HER2-negative early breast cancer patients. Oncotarget (2017) 8(24):38706–16. 10.18632/oncotarget.16313 PMC5503565 28404972

[6] Wu J Gao W Chen X Fei C Lin L Chen W. Prognostic value of the 21-gene recurrence score in ER-positive, HER2-negative, node-positive breast cancer was similar in node-negative diseases: a single-center study of 800 patients. Front Med (2020). 10.1007/s11684-020-0738-0 33367943

[7] Salmon H Remark R Gnjatic S Merad M. Host tissue determinants of tumour immunity. Nat Rev Cancer (2019) 19(4):215–27. 10.1038/s41568-019-0125-9 PMC7787168 30867580

[8] Picon-Ruiz M Morata-Tarifa C Valle-Goffin JJ Friedman ER Slingerland JM. Obesity and adverse breast cancer risk and outcome: Mechanistic insights and strategies for intervention. CA Cancer J Clin (2017) 67(5):378–97. 10.3322/caac.21405 PMC5591063 28763097

[9] Bandera EV John EM. Obesity, Body Composition, and Breast Cancer: An Evolving Science. JAMA Oncol (2018) 4(6):804–5. 10.1001/jamaoncol.2018.0125 29621383

[10] Renehan AG Tyson M Egger M Heller RF Zwahlen M. Body-mass index and incidence of cancer: a systematic review and meta-analysis of prospective observational studies. Lancet (2008) 371(9612):569–78. 10.1016/S0140-6736(08)60269-X 18280327

[11] Kawai M Malone KE Tang MT Li CI. Height, body mass index (BMI), BMI change, and the risk of estrogen receptor-positive, HER2-positive, and triple-negative breast cancer among women ages 20 to 44 years. Cancer (2014) 120(10):1548–56. 10.1002/cncr.28601 PMC4013221 24500704

[12] Muniz J Kidwell KM Henry NL. Associations between metabolic syndrome, breast cancer recurrence, and the 21-gene recurrence score assay. Breast Cancer Res Treat (2016) 157(3):597–603. 10.1007/s10549-016-3846-4 27271766 PMC5095927

[13] Sestak I Dowsett M Ferree S Baehner FL Cuzick J. Retrospective analysis of molecular scores for the prediction of distant recurrence according to baseline risk factors. Breast Cancer Res Treat (2016) 159(1):71–8. 10.1007/s10549-016-3868-y PMC5010586 27447876

[14] Ministry of Health of the People’s Republic of China. The guidelines for prevention and control of overweight and obesity in Chinese adults. Beijing, China: People’s Medical Publishing House (2006).

[15] Wang F Liu L Cui S Tian F Fan Z Geng C. Distinct Effects of Body Mass Index and Waist/Hip Ratio on Risk of Breast Cancer by Joint Estrogen and Progestogen Receptor Status: Results from a Case-Control Study in Northern and Eastern China and Implications for Chemoprevention. Oncologist (2017) 22(12):1431–43. 10.1634/theoncologist.2017-0148 PMC5728030 28912152

[16] Hammond ME Hayes DF Dowsett M Allred DC Hagerty KL Badve S. American Society of Clinical Oncology/College Of American Pathologists guideline recommendations for immunohistochemical testing of estrogen and progesterone receptors in breast cancer. J Clin Oncol (2010) 28(16):2784–95. 10.1200/JCO.2009.25.6529 PMC2881855 20404251

[17] Goldhirsch A Ingle JN Gelber RD Coates AS Thurlimann B Senn HJ. Thresholds for therapies: highlights of the St Gallen international expert consensus on the primary therapy of early breast cancer 2009. Ann Oncol (2009) 20:1319–29. 10.1093/annonc/mdp322 PMC2720818 19535820

[18] Goldhirsch A Winer EP Coates AS Gelber RD Piccart-Gebhart M THurlimann B. Personalizing the treatment of women with early breast cancer: highlights of the St Gallen International Expert Consensus on the Primary Therapy of Early Breast Cancer 2013. Ann Oncol (2013) 24(9):2206–23. 10.1093/annonc/mdt30 PMC3755334 23917950

[19] Wolff AC Hammond MEH Allison KH Harvey BE Mangu PB Bartlett JMS. Human Epidermal Growth Factor Receptor 2 Testing in Breast Cancer: American Society of Clinical Oncology/College of American Pathologists Clinical Practice Guideline Focused Update. J Clin Oncol (2018) 36(20):2105–22. 10.1200/JCO.2018.77.8738 29846122

[20] Hudis CA Barlow WE Costantino JP Gray RJ Pritchard KI Chapman JA. Proposal for standardized definitions for efficacy end points in adjuvant breast cancer trials: the STEEP system. J Clin Oncol (2007) 25(15):2127–32. 10.1200/JCO.2006.10.3523 17513820

[21] Murphy WJ Longo DL. The Surprisingly Positive Association Between Obesity and Cancer Immunotherapy Efficacy. JAMA (2019) 321(13):1247–8. 10.1001/jama.2019.0463 30882850

[22] Dignam JJ Wieand K Johnson KA Johnson KA Fisher B Xu L. Obesity, tamoxifen use, and outcomes in women with estrogen receptor-positive early-stage breast cancer. J Natl Cancer Inst (2003) 95(19):1467–76. 10.1093/jnci/djg060 PMC4676737 14519753

[23] Sparano JA Wang M Zhao F Stearns V Martino S Ligibel JA. Obesity at diagnosis is associated with inferior outcomes in hormone receptor-positive operable breast cancer. Cancer (2012) 118(23):5937–46. 10.1002/cncr.27527 PMC3586227 22926690

[24] Niraula S Ocana A Ennis M Goodwin PJ. Body size and breast cancer prognosis in relation to hormone receptor and menopausal status: a meta-analysis. Breast Cancer Res Treat (2012) 134(2):769–81. 10.1007/s10549-012-2073-x 22562122

[25] Cespedes Feliciano EM Kwan ML Kushi LH Chen WY Weltzien EK Castillo AL. Body mass index, PAM50 subtype, recurrence, and survival among patients with nonmetastatic breast cancer. Cancer (2017) 123(13):2535–42. 10.1002/cncr.30637 PMC5474169 28295245

[26] Makama M Drukker CA Rutgers EJT Slaets L Cardoso F Rookus MA. An association study of established breast cancer reproductive and lifestyle risk factors with tumour subtype defined by the prognostic 70-gene expression signature (MammaPrint((R))). Eur J Cancer (2017) 75:5–13. 10.1016/j.ejca.2016.12.024 28214658

[27] Key TJ Appleby PN Reeves GK Roddam A Dorgan JF Longcope C. Body mass index, serum sex hormones, and breast cancer risk in postmenopausal women. J Natl Cancer Institute (2003) 95(16):1218–26. 10.1093/jnci/djg022 12928347

[28] Canchola AJ Anton-Culver H Bernstein L Clarke CA Henderson K Ma H. Body size and the risk of postmenopausal breast cancer subtypes in the California Teachers Study cohort. Cancer Causes Control CCC (2012). 10.1007/s10552-012-9897-x PMC3366039 22286371

[29] Kwan ML Kroenke CH Sweeney C Bernard PS Weltzien EK Castillo A. Association of high obesity with PAM50 breast cancer intrinsic subtypes and gene expression. BMC Cancer (2015) 15:278. 10.1186/s12885-015-1263-4 25884832 PMC4403771

