# Supplementary figures and images for: Comprehensive Association Analysis of 21-Gene Recurrence Score and Obesity in Chinese Breast Cancer Patients
Source: Front Oncol. 2021 Mar 26;11:619840. doi: 10.3389/fonc.2021.619840 (PMC8032994; doi:10.3389/fonc.2021.619840)

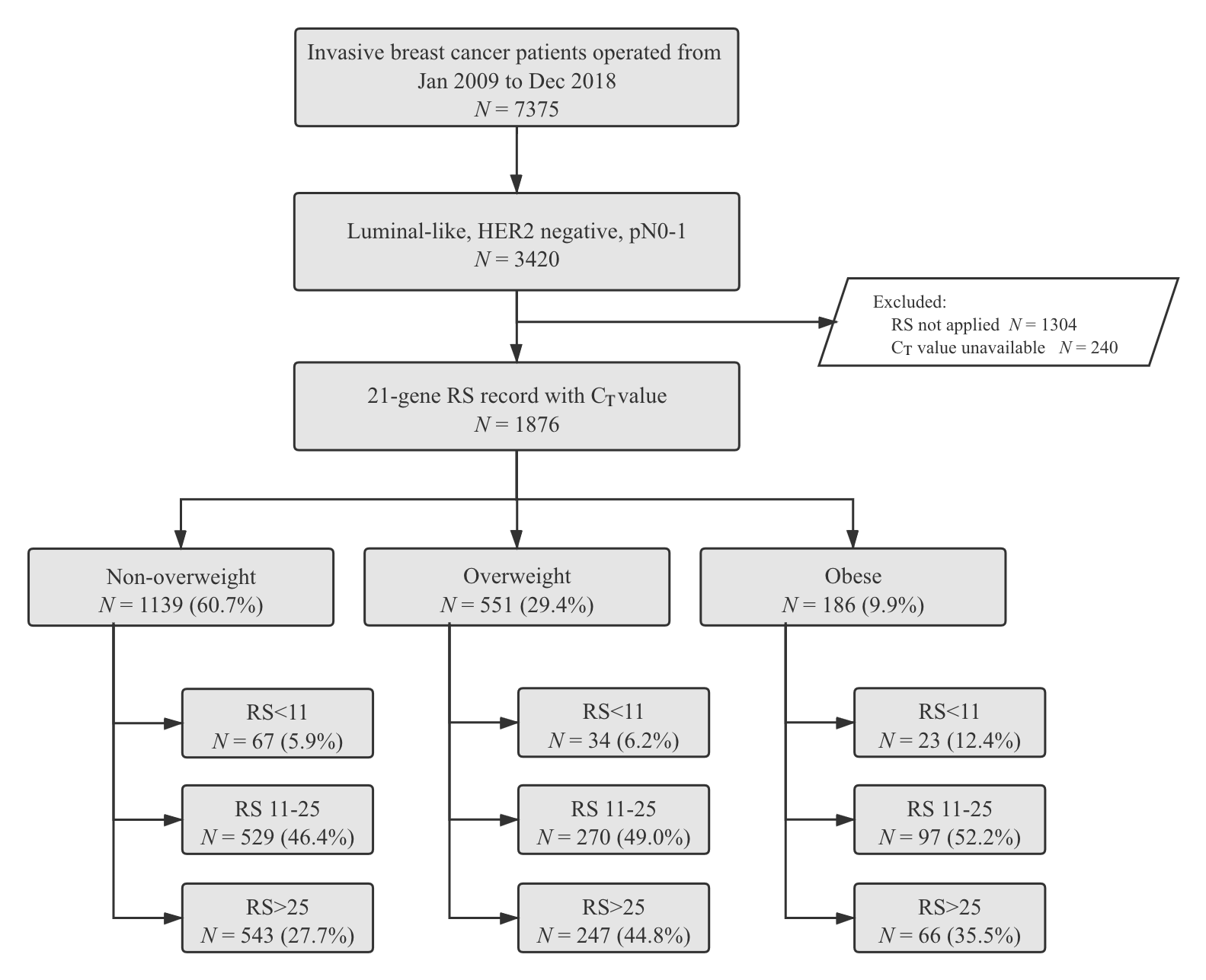

Supplement: Supplementary Figure S1 — Study population flowchart. HER2, human epidermal growth factor receptor 2; RS, recurrence score; CT, cycle threshold. [file Image_1.jpeg]

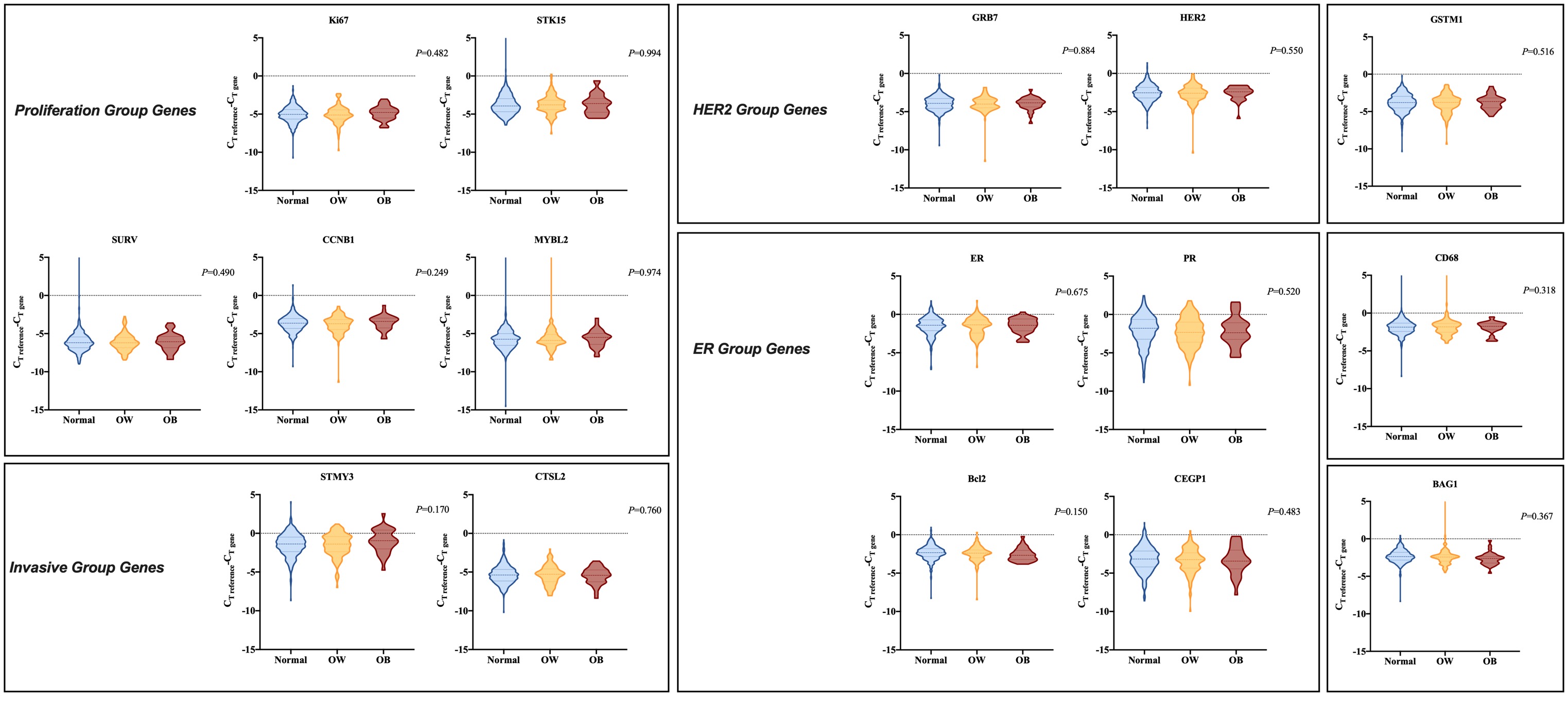

Supplement: Supplementary Figure S2 — Comparison of gene expression in 21-gene RS panel by BMI status in premenopausal population. The violin plots refer to the expression of a certain gene. The dashed lines and the dotted lines refer to the median and quartiles, respectively. BMI, body mass index; CT, cycle threshold; OW, overweight; OB, obese; HER2, human epidermal growth factor receptor 2; ER, estrogen receptor. [file Image_2.jpeg]

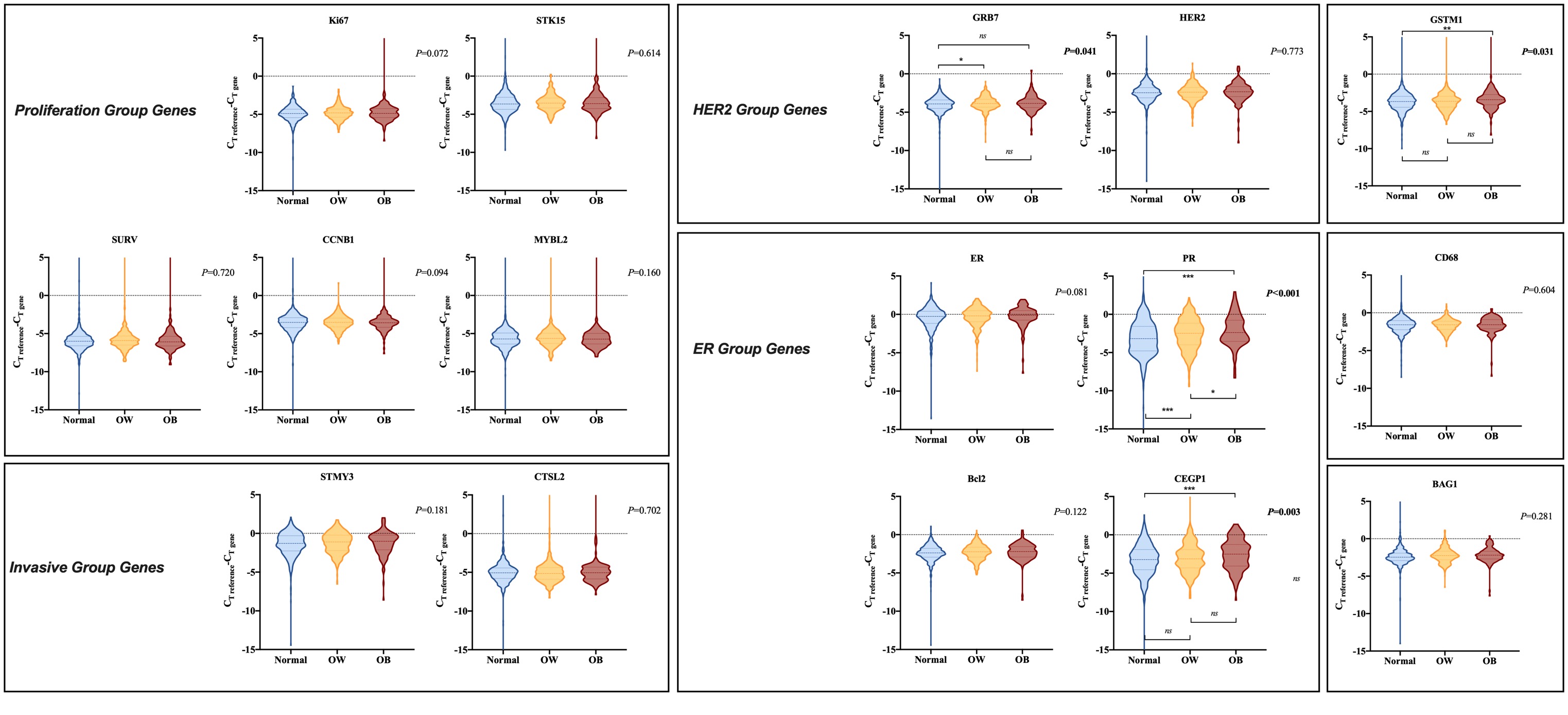

Supplement: Supplementary Figure S3 — Comparison of gene expression in 21-gene RS by BMI status in postmenopausal population. The violin plots refer to the expression of a certain gene. The dashed lines and the dotted lines refer to the median and quartiles, respectively. BMI, body mass index; CT, cycle threshold; OW, overweight; OB, obese; HER2, human epidermal growth factor receptor 2; ER, estrogen receptor. [file Image_3.jpeg]

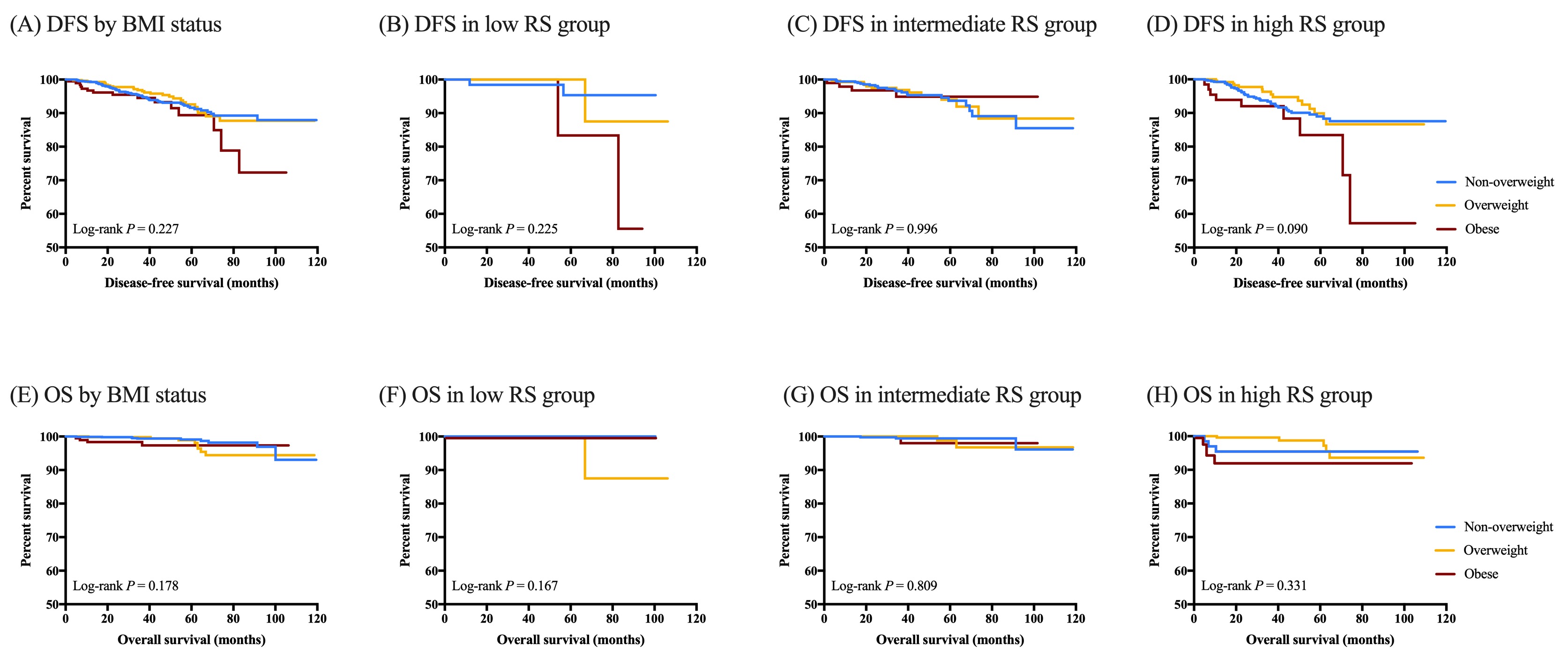

Supplement: Supplementary Figure S4 — Clinical outcomes of breast cancer patients with different BMI statuses stratified by 21-gene RS category. DFS in (A) whole, (B) low risk, (C) intermediate risk, and (D) high risk population; OS in (E) whole, (F) low risk, (G) intermediate risk, and (H) high risk population. Navy, yellow and red lines refer to non-overweight, overweight and obese group, respectively. DFS, disease-free survival; OS, overall survival; BMI, body mass index; RS, recurrence score. [file Image_4.jpeg]

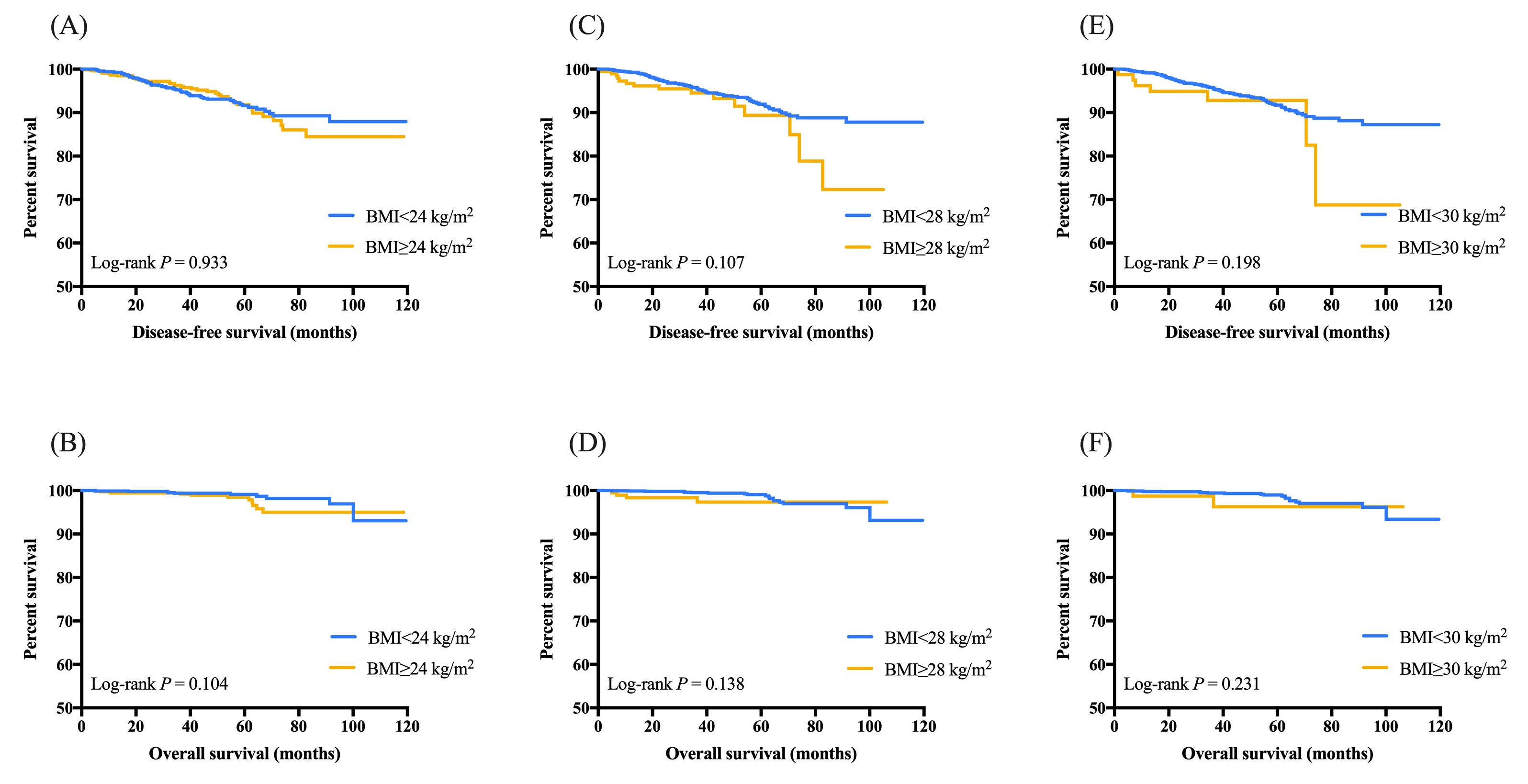

Supplement: Supplementary Figure S5 — Clinical outcomes of breast cancer patients with different BMI cutoffs. Disease-free survival and overall survival (A, B) whole, (B) low risk, (C) intermediate risk, and (D) high risk population; OS in (E) whole, (F) low risk, (G) intermediate risk, and (H) high risk population. [file Image_5.jpeg]
