# Supplementary material for: Comprehensive Association Analysis of 21-Gene Recurrence Score and Obesity in Chinese Breast Cancer Patients
Source: Front Oncol. 2021 Mar 26;11:619840. doi: 10.3389/fonc.2021.619840 (PMC8032994; doi:10.3389/fonc.2021.619840)
Supplement: Supplementary file 6 [file DataSheet_1.docx]

**Supplementary Table S1. 21-gene expression in study population (*N* = 1876)**

| **Gene** | **Mean** | **Median** | **SD** |
| --- | --- | --- | --- |
| C_T_ *GRB7* | 32.99 | 32.91 | 1.28 |
| C_T_ *HER2* | 31.54 | 31.62 | 1.59 |
| C_T_ *ER* | 29.87 | 29.90 | 1.60 |
| C_T_ *PR* | 31.67 | 31.84 | 2.19 |
| C_T_ *Bcl2* | 31.43 | 31.14 | 1.34 |
| C_T_ *CEGP1* | 32.20 | 31.96 | 2.11 |
| C_T_ *CCNB1* | 32.64 | 32.80 | 1.85 |
| C_T_ *Ki67* | 33.98 | 33.92 | 1.54 |
| C_T_ *MYBL2* | 34.53 | 34.86 | 3.05 |
| C_T_ *STK15* | 32.62 | 32.78 | 2.05 |
| C_T_ *SURV* | 34.94 | 35.11 | 2.40 |
| C_T_ *CTSL2* | 34.20 | 34.32 | 1.84 |
| C_T_ *STMY3* | 30.44 | 29.97 | 1.90 |
| C_T_ *CD68* | 30.71 | 30.89 | 2.15 |
| C_T_ *GSTM1* | 32.76 | 32.87 | 2.02 |
| C_T_ *BAG1* | 31.36 | 31.33 | 1.67 |
| C_T_ *β-actin* | 24.90 | 24.91 | 1.50 |
| C_T_ *GAPDH* | 27.94 | 27.93 | 1.59 |
| C_T_ *GUS* | 31.75 | 31.87 | 1.93 |
| C_T_ *RPLPO* | 29.54 | 29.90 | 1.84 |
| C_T_ *TFRC* | 31.19 | 30.97 | 1.60 |
| HER2 group score | 8.06 | 8.00 | 0.27 |
| ER group score | 7.97 | 8.00 | 1.22 |
| Proliferation group score | 6.69 | 6.50 | 1.00 |
| Invasion group score | 7.03 | 7.00 | 1.28 |

Abbreviations: SD, standard deviation; C_T_, cycle threshold; HER2, human epidermal growth factor receptor 2; ER, estrogen receptor.

**Supplementary Table S2. Univariate analysis of impact factors for DFS and OS (*N*=1876)**

| Factor | All patients  Log-rank *P* value | |  | Non-overweight  Log-rank *P* value | |  | Overweight  Log-rank *P* value | |  | Obese  Log-rank *P* value | |
| --- | --- | --- | --- | --- | --- | --- | --- | --- | --- | --- | --- |
|  | DFS | OS |  | DFS | OS |  | DFS | OS |  | DFS | OS |
| Age | 0.693 | 0.003 |  | 0.270 | 0.011 |  | 0.825 | 0.226 |  | 0.528 | 0.152 |
| Menstruation | 0.469 | 0.019 |  | 0.385 | 0.030 |  | 0.661 | 0.203 |  | 0.433 | 0.684 |
| BMI | 0.227 | 0.178 |  | / | / |  | / | / |  | / | / |
| Histology | 0.087 | 0.235 |  | 0.002 | 0.023 |  | 0.317 | 0.989 |  | 0.584 | 0.495 |
| Tumor grade | 0.005 | 0.212 |  | 0.036 | 0.580 |  | 0.076 | 0.191 |  | 0.712 | 0.734 |
| Tumor size | 0.001 | 0.363 |  | 0.008 | 0.653 |  | 0.074 | 0.761 |  | 0.212 | 0.039 |
| ALN | 0.543 | 0.663 |  | 0.588 | 0.908 |  | 0.716 | 0.943 |  | 0.860 | 0.603 |
| ER | 0.048 | 0.015 |  | 0.787 | 0.170 |  | 0.006 | 0.006 |  | 0.010 | 0.642 |
| PR | 0.127 | 0.204 |  | 0.677 | 0.666 |  | 0.342 | 0.038 |  | 0.008 | 0.242 |
| Ki-67 | 0.001 | 0.154 |  | 0.081 | 0.665 |  | <0.001 | 0.154 |  | 0.567 | 0.044 |
| MS | 0.003 | 0.172 |  | 0.065 | 0.462 |  | 0.003 | 0.039 |  | 0.652 | 0.125 |
| RS category | 0.001 | 0.194 |  | 0.046 | 0.404 |  | 0.558 | 0.530 |  | 0.114 | 0.219 |
| Adjuvant CT | 0.143 | 0.312 |  | 0.205 | 0.283 |  | 0.228 | 0.909 |  | 0.871 | 0.502 |
| Adjuvant RT | 0.872 | 0.043 |  | 0.946 | 0.127 |  | 0.848 | 0.290 |  | 0.902 | 0.452 |
| Adjuvant ET | 0.002 | 0.070 |  | 0.029 | 0.233 |  | 0.108 | 0.028 |  | 0.076 | 0.766 |

Abbreviations: RFS, recurrence-free survival; OS, overall survival; ALN, axillary lymph node; BMI, body mass index; ER, estrogen receptor; PR, progesterone receptor; MS, molecular subtype; RS, recurrence score; CT, chemotherapy; RT, radiotherapy; ET, endocrine therapy.
